# Dataset for comparative analysis of precision metagenomics and traditional methods in urinary tract infection diagnostics

**DOI:** 10.1016/j.dib.2025.111339

**Published:** 2025-01-30

**Authors:** Rob E. Carpenter, Sadia Almas, Vaibhav K. Tamrakar, Rahul Sharma

**Affiliations:** a Department of Research, Advanta Genetics, Tyler, TX 75703, United States; b Soules College of Business, The University of Texas at Tyler, 3900 University Boulevard, Tyler, TX 75799, United States; c Department of Research, Scienetix, Tyler, TX 75703, United States; d Department of Research, RetroBiotech and Research Pvt. Ltd, Jaipur, RJ 302017, India

**Keywords:** Precision metagenomics, Urinary tract infection, PCR, Microbial culture, Next-generation sequencing, Uropathogen identification

## Abstract

This study presents a comprehensive dataset comparing three diagnostic methodologies—microbial culture, polymerase chain reaction (PCR), and precision metagenomics (precision metagenomics)—for the detection and classification of uropathogens in urine samples from patients with suspected urinary tract infections (UTIs). While microbial culture remains the gold standard for UTI diagnosis, it has limitations in sensitivity, particularly for fastidious or non-culturable microorganisms. PCR offers higher sensitivity but is restricted to pre-targeted organisms, limiting its diagnostic range. Precision Metagenomics, a target-agnostic sequencing method, provides a more inclusive approach by enabling the identification of a broad spectrum of pathogens, including bacteria, viruses, fungi, and parasites, without prior knowledge of the organisms. The dataset includes 47 urine samples, each analyzed by microbial culture, PCR, and precision metagenomics, followed by bioinformatic classification using the Explify® platform. precision metagenomics identified significantly more uropathogens (62 distinct organisms) compared to PCR (19 organisms) and microbial culture (13 organisms), with 98 % of samples testing positive for polymicrobial infections via precision metagenomics. The precision metagenomics method demonstrated superior diagnostic yield by detecting pathogens that were missed by both microbial culture and PCR, particularly in culture-negative and PCR-negative cases. This dataset holds substantial reuse potential for further research into the microbiome of urinary tract infections, pathogen discovery, antimicrobial resistance studies, and the development of more accurate diagnostic models for UTI management. By offering insights into both polymicrobial infections and rare pathogens, this dataset supports the advancement of diagnostic strategies for complex and chronic UTIs.

Specifications Table

**Table:** 

Subject	Molecular Biology, Infectious Diseases
Specific subject area	Urinary Tract Infections Diagnostics.
Type of data	Tables, Figures, Raw Data, Processed Data
Data collection	Processed, Analyzed Data were obtained from 47 urine samples collected from patients with clinically suspected UTIs. Each sample underwent a comprehensive diagnostic evaluation using three methodologies: microbial culture, PCR, and precision metagenomics. Microbial culture was used to identify culturable uropathogens, while PCR targeted 28 specific uropathogens, including bacteria and fungi. Precision Metagenomics employed a target-agnostic hybridization capture approach, enabling the detection of a broader spectrum of microorganisms, including fastidious and non-culturable organisms. Precision metagenomics data were analyzed using the Explify® bioinformatics platform, which provided both qualitative identification and phenotypic classification of detected microorganisms. Each microorganism was categorized into one of four phenotypic groups, ranging from common contaminants to pathogens with high clinical relevance, based on their pathogenic potential and association with UTI.
Data source location	Institution: Advanta Genetics, LLC Address: 10935 CR 159, Tyler, Texas 75703 Country: United States Latitude and longitude: 31.9686 ° N, 99.9018 ° W
Data accessibility	Repository: NCBI Sequence Read Archive Data Identification Number: PRJNA986135 Direct URL: https://www.ncbi.nlm.nih.gov/sra/?term=PRJNA986135 Instructions for access: The dataset is openly accessible through the NCBI SRA repository.
Related research article	Almas, S., Carpenter, R. E., Rowan, C., Tamrakar, V. K., Bishop, J., & Sharma, R. (2023). Advantage of Precision Metagenomics for Urinary Tract Infection Diagnostics. *Frontiers in Cellular and Infection Microbiology*, 13, 1221289. DOI: 10.3389/fcimb.2023.1221289.

## 1 Value of the Data

• The dataset provides a comparative analysis of three diagnostic methods—microbial culture, PCR, and precision metagenomics—for urinary tract infection diagnostics [1], showcasing the enhanced detection capabilities of precision metagenomics [2].
• Researchers can reuse the dataset to further investigate polymicrobial infections and explore the role of hybridization capture-based sequencing in diagnostics.
• The dataset supports the development of improved diagnostic models for complex UTI cases by providing comprehensive bioinformatic analyses of microbial populations.
• It can contribute to studies on antimicrobial resistance [3] and the clinical impact of using advanced diagnostic tools like precision metagenomics in patient care.
• The dataset is a valuable resource for validating new bioinformatic tools and methods for classifying pathogenic organisms in UTI cases.

## 2 Background

The motivation behind this dataset stems from the critical need for more advanced and comprehensive diagnostic methods that can bridge the gap left by traditional techniques [4]. Precision metagenomics, a target-agnostic sequencing approach, has emerged as a promising alternative due to its ability to identify a wide range of pathogens, including bacteria, viruses, fungi, and parasites, without prior knowledge of their presence [5]. By sequencing all microbial DNA within a sample, precision metagenomics allows for a more thorough analysis of the microbial ecosystem in the urinary tract, including the urobiome, which may play a significant role in recurrent and chronic UTIs [6].

**Fig. 1:**
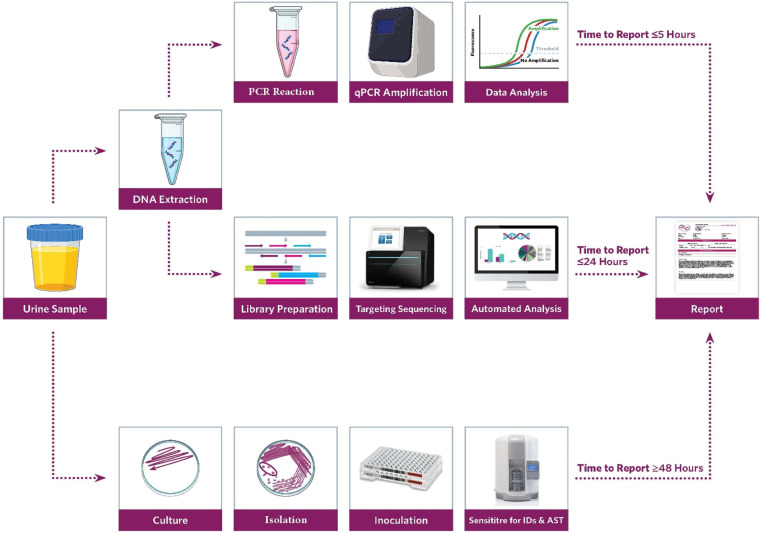
Precision Metagenomics Workflow for Diagnosing Urinary Tract Infections (UTIs) – This diagram outlines the detailed steps involved in the precision metagenomics process, from sample collection to bioinformatic analysis using the Explify® platform for pathogen identification and classification.

This study was designed to address these limitations by comparing the diagnostic yields of microbial culture, PCR, and precision metagenomics, using 47 urine samples from patients with clinically suspected UTIs. The goal was to evaluate whether a precision metagenomics workflow (Fig. 1) could enhance the detection of uropathogens, including those missed by traditional methods, and provide a more accurate and comprehensive understanding of the microbial diversity in UTI cases. By leveraging the bioinformatic capabilities of the Explify® platform [7], the study also aimed to classify pathogens phenotypically, offering insights into their potential clinical relevance and pathogenicity. The ultimate objective was to assess the feasibility of using precision metagenomics as a routine diagnostic tool in clinical practice, with the potential to improve patient outcomes through earlier and more accurate diagnosis and tailored treatment strategies.

## 3 Data Description

The dataset generated from this study [8] encompasses a comprehensive comparison of three diagnostic techniques—microbial culture, PCR, and precision metagenomics—applied to 47 urine samples collected from patients with suspected UTIs. The dataset contains the following key components:

### 3.1 Organism identification results from microbial culture, PCR, and precision metagenomics

Each of the 47 urine samples was analyzed using microbial culture, PCR, and precision metagenomics, with the results for organism identification meticulously recorded for each method. The dataset captures detailed information about which specific pathogens were detected by each technique, including common uropathogens such as *Escherichia coli* and Enterococcus faecalis, as well as rarer or non-culturable organisms detected solely by precision metagenomics. This allows for a comparative view of the sensitivity and scope of each method in identifying uropathogens in clinical settings [9,10].

### 3.2 Phenotypic classification of identified organisms

Organisms identified through precision metagenomics were further categorized based on their potential pathogenicity, using a four-tier phenotypic classification system provided by the Explify® platform. This classification provides a structured view of the clinical significance of each organism, aiding in the understanding of which organisms are likely contributing to infection versus those present as background flora. Organisms were grouped as follows:

• Group 0: Organisms unlikely to cause UTIs, often considered contaminants or part of the normal microbiota.
• Group 1: Organisms occasionally implicated in UTIs but more commonly part of the natural urobiome or considered colonizers.
• Group 2: Microorganisms frequently associated with UTIs and considered likely to contribute to infection in certain clinical contexts.
• Group 3: Pathogens routinely implicated in UTIs with high pathogenic potential and clinical relevance.

### 3.3 Detailed bioinformatic analysis results using the Explify® platform

Precision metagenomics data were processed through the Explify® platform, which provides both qualitative and quantitative microbial profiles for each sample. This bioinformatic analysis includes sequencing depth, read counts, and relative abundance of microorganisms, offering insights into the microbial ecology within each sample. The data reflect the platform's ability to differentiate between organisms with similar genetic sequences and provide an estimate of each pathogen's abundance. This level of detail is particularly useful for understanding the potential role of microbial interactions in polymicrobial infections and for assessing pathogen load, which may be relevant for clinical management decisions.

### 3.4 Summary tables comparing the diagnostic yield of each method

The dataset includes summary tables that facilitate comparison between microbial culture, PCR, and precision metagenomics in terms of the number and types of organisms detected. These tables highlight the diagnostic yield of each method, illustrating the enhanced discovery power of precision metagenomics, especially in identifying polymicrobial infections and non-culturable or fastidious organisms that were missed by other methods. Additionally, the tables quantify how often each method detected organisms exclusively, providing a clear picture of the strengths and limitations of each technique.

## 4 Experimental Design, Materials and Methods

### 4.1 Materials and methods

#### 4.1.1 Sample collection and handling

Urine specimens were collected under sterile conditions following standard clinical protocols to prevent contamination. All samples were transported to the Advanta Genetics Laboratory in Tyler, TX, where they were processed within 24 h and stored at 4 °C prior to analysis.

#### 4.1.2 Microbial culture procedures

For traditional microbial culture analysis, 1 µl of each urine sample was inoculated onto Spectra UTI chromogenic biplates (ThermoFisher, Carlsbad, CA). These plates facilitate the differentiation and presumptive identification of uropathogens based on colony color and morphology. The plates were incubated aerobically at 37 °C for an initial period of 24 h, with an additional 24 h if no growth was observed during the first incubation. Microbial colonies were quantified in terms of colony-forming units (CFUs), with results recorded in logarithmic scales (<10⁴, 10⁴–10⁵, or >10⁵ CFU/ml). Only counts above 10⁴ CFU/ml were considered clinically significant. Colony identification was aided by Gram staining, chromogenic reactions, and further confirmed through the Sensititre™ ARIS HiQ™ System (ThermoFisher).

#### 4.1.3 PCR testing

To detect specific uropathogens, each urine sample was subjected to PCR analysis targeting 28 microorganisms (24 bacteria and 4 fungi). DNA was extracted using an automated system, and 2.5 µl of the extracted material was mixed with 7.5 µl of master mix containing pathogen-specific primers and TaqMan® probes. The PCR reactions were performed using a Roche Light Cycler® 384-well instrument under the following cycling conditions: 95 °C for 3 mins for initial denaturation, followed by 40 cycles of 95 °C for 5 ss and 60 °C for 30 ss for amplification. Positive detection was defined as a cycle threshold (Ct) value of ≤35 with an accompanying sigmoid amplification curve, indicating the presence of targeted pathogens.

#### 4.1.4 Nucleic acid extraction for precision metagenomics analysis

For precision metagenomics analysis, urine samples were homogenized by vortexing for 10 s before transferring 500 µl into 2-ml safe-lock tubes containing zirconium oxide beads and proteinase K. The samples were lysed using a TissueLyser (Qiagen Inc., Hilden, Germany) at 30 Hz for 5 min to break down cellular structures. A 150-µl aliquot of the lysed sample was then combined with internal control (IC) DNA in a 96-well plate and subjected to automated nucleic acid extraction on the Roche MagNA Pure 96 platform using the DNA and Viral NA Small Volume Kit. The final elution volume was 100 µl per sample. The extraction process was verified by spiking each sample with synthetic T7 bacteriophage DNA, which was detected by subsequent PCR testing to confirm successful extraction.

#### 4.1.5 Precision metagenomics library preparation and sequencing

For precision metagenomics sequencing, libraries were prepared using the Illumina DNA Prep with Enrichment Tagmentation Kit (Illumina, San Diego, CA). This process involved tagmentation and adapter ligation of extracted DNA, followed by the hybridization capture of microbial target sequences using a custom capture panel that focused on 135 bacterial, 35 viral, 14 fungal, and 7 parasitic organisms. The enriched libraries were pooled in triplicate and hybridized to UTI Pathogen ID-AMR probes at 58 °C for 90 min following initial denaturation at 95 °C. Captured libraries were then amplified for 18 cycles and purified using AmPureXP beads (Beckman Coulter).

Libraries were quantified using a Qubit 2.0 Fluorometer (Invitrogen, Waltham, MA), and the size distribution of the DNA fragments was checked with an Agilent 5200 Fragment Analyzer (Agilent, Austin, TX). The normalized libraries were loaded onto an Illumina MiniSeq® instrument and sequenced using paired-end 75-bp reads. Each sample was required to generate at least 0.5 million reads to ensure a robust microbial profile.

#### 4.1.6 Bioinformatic analysis using explify®

The sequencing data obtained from the Illumina MiniSeq® were processed using the Explify® Urinary Pathogen ID/AMR Panel (UPIP) bioinformatic platform. Sequencing reads were demultiplexed based on sample-specific barcodes, and only samples passing quality control checks were included in the analysis. Explify® compared sequencing reads to a curated reference database of microbial genomes and classified detected organisms into four phenotypic groups based on their potential pathogenicity (Tables 1–3):

• Group 0: Common contaminants or organisms unlikely to cause UTI.
• Group 1: Normal flora with the potential to cause UTI under certain conditions.
• Group 2: Organisms frequently associated with UTI, though sometimes present as colonizers.
• Group 3: Pathogens are regularly implicated in UTI cases.

**Table 1:** Microorganism detection in Phenotypic Group 1 across diagnostic methods.

Testing Category	Organism Count	Detected Organisms
Unique to NGS	12	*Acinetobacter pittii, Actinobaculum massiliense, Actinotignum sanguinis, Aerococcus christensenii, Aerococcus urinae, Alloscardovia omnicolens, Anaerococcus lactolyticus, Corynebacterium aurimucosum, Corynebacterium glucuronolyticum, Finegoldia magna (Peptostreptococcus magnus), Staphylococcus simulans, Staphylococcus warneri*
Unique to Culture	4	*Enterococcus faecalis, E. coli, Klebsiella oxytoca, Proteus mirabilis*
Unique to PCR	5	*Actinotignum schaalii, Candida albicans, Candida glabrata, Candida parapsilosis, Citrobacter freundii/braakii/koseri*
Overlap: All Three Methods	3	*Enterococcus faecalis, E. coli, Proteus mirabilis*
Overlap: mNGS + PCR	3	*Bacteroides fragilis, Klebsiella oxytoca, Staphylococcus aureus*
Overlap: mNGS + Culture	1	*Staphylococcus epidermidis*
Overlap: Culture + PCR	2	*Citrobacter freundii, Enterococcus faecalis*

**Table 2:** Microorganism detection in Phenotypic Group 2 across diagnostic methods.

Testing Category	Organism Count	Detected Organisms
Unique to NGS	20	*Bifidobacterium breve, Candida glabrata (Nakaseomyces glabrata), Corynebacterium coyleae, Corynebacterium pseudogenitalium, Enterobacter cloacae complex, Enterococcus raffinosus, Facklamia hominis, Klebsiella quasipneumoniae, Klebsiella variicola, Mobiluncus curtisii, Peptostreptococcus anaerobius, Porphyromonas asaccharolytica, Prevotella timonensis, Propionimicrobium lymphophilum, Providencia stuartii, Rothia kristinae (Kocuria kristinae), Salmonella enterica, Serratia marcescens, Streptococcus anginosus, Streptococcus constellatus*
Unique to Culture	5	*Acinetobacter baumannii, Enterobacter aerogenes, Enterobacter cloacae, Klebsiella pneumoniae, Lactobacillus* species
Unique to PCR	5	*Enterococcus faecium, E. coli, Klebsiella aerogenes, Klebsiella pneumoniae, Prevotella bivia*
Overlap: All Three Methods	0	None Detected
Overlap: mNGS + PCR	4	*Enterococcus faecium, Klebsiella pneumoniae, Morganella morganii, Streptococcus agalactiae*
Overlap: mNGS + Culture	0	None Detected
Overlap: Culture + PCR	2	*Enterococcus faecium, Klebsiella pneumoniae*

**Table 3:** Microorganism detection in Phenotypic Group 3 across diagnostic methods.

Testing category	Organism Count	Detected Organisms
Unique to NGS	20	BK polyomavirus, Epstein-Barr virus (EBV), Human adenovirus B, Human papillomavirus type 51 (HPV 51; High-risk), Human papillomavirus type 55/44 (HPV 55/44; Low-risk), Human papillomavirus type 56 (HPV 56; High-risk), Human papillomavirus type 68 (HPV 68; High-risk), JC polyomavirus, Staphylococcus haemolyticus, Staphylococcus hominis, Streptococcus intermedius, Trichomonas vaginalis, Ureaplasma parvum
Unique to Culture	1	*Staphylococcus aureus*
Unique to PCR	4	*Candida parapsilosis, Enterobacter cloaca, Streptococcus agalactiae, Candida albicans*
Overlap: All Three Methods	0	None detected.
Overlap: mNGS + PCR	1	*Streptococcus agalactiae*
Overlap: mNGS + Culture	0	None detected.
Overlap: Culture + PCR	0	None detected.

Our downstream analysis utilized the Explify® bioinformatics platform which classified each microbe into phenotypic groups 0–3^1^—applying an escalating order of potential pathogenicity. Human papillomavirus and *Trichomonas vaginalis* were the only microorganisms classified in phenotypic group-0 because both are common etiological agents of sexually transmitted infection, not UTI. Microorganisms classified in phenotypic group-1 are frequently considered part of the normal flora but with the potential for associated UTI diseases in certain clinical manifestations. In addition to identification, the platform provided quantitative data on organism abundance, though antimicrobial resistance profiling and quantitative reporting were beyond the scope of this study.

#### 4.1.7 Comparative performance analysis

**Fig. 2:**
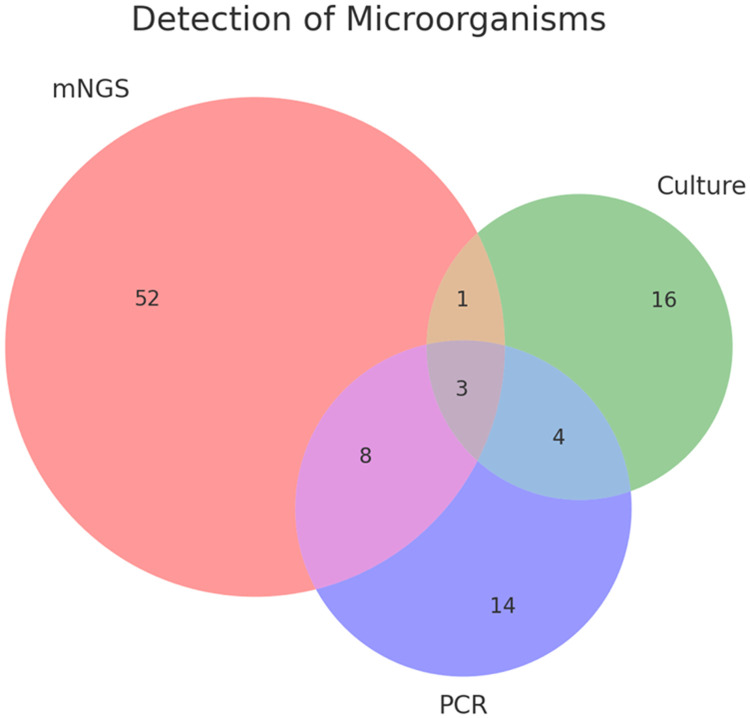
The Venn diagram illustrates the detection of microorganisms using three methods: metagenomics next-generation sequencing (mNGS), culture, and polymerase chain reaction (PCR). The diagram shows that 52 microorganisms were detected exclusively by mNGS, 16 exclusively by culture, and 14 exclusively by PCR. Additionally, 4 microorganisms were detected by both culture and PCR but not by mNGS, 8 were detected by both mNGS and PCR but not by culture, and 1 was detected by both mNGS and culture but not by PCR. Notably, 3 microorganisms were concordantly detected by all three methods. This visualization highlights the complementary nature of these methods in microbial detection.

The dataset was analyzed to assess the concordance and discordance between microbial culture, PCR, and precision metagenomics in identifying uropathogens (Table 4). The ability of precision metagenomics to detect polymicrobial infections, fastidious organisms, and pathogens that are non-culturable was compared to PCR and traditional culture techniques (Fig. 2). Specific attention was given to cases where precision metagenomics identified pathogens not detected by the other methods.

#### 4.1.8 Limitations and challenges

The PCR results presented in these tables are constrained by the scope of validated organisms included in the specific PCR assay utilized in this study. As a result, the detection of microorganisms is limited to those for which the assay was designed and validated. Additionally, the culture results are inherently restricted to bacterial organisms, as the study employed culture techniques optimized exclusively for the growth of bacterial species. These limitations highlight the need for complementary diagnostic methods, such as metagenomics mNGS, to achieve a broader and more comprehensive detection of pathogens (Table 2).

**Table 4:** Microorganism detection across diagnostic methods: Unique and overlapping categories.

Testing Category	Organism Count	Detected Organisms
Unique to NGS	52	Acinetobacter pittii, Actinobaculum massiliense, Actinotignum sanguinis, Actinotignum sanguinis (Actinobaculum schaalii), Aerococcus christensenii, Aerococcus urinae, Alloscardovia omnicolens, Anaerococcus lactolyticus, Atopobium vaginae, BK polyomavirus, Bifidobacterium breve, Candida glabrata (Nakaseomyces glabrata), Citrobacter freundii complex, Corynebacterium aurimucosum, Corynebacterium coyleae, Corynebacterium glucuronolyticum, Corynebacterium pseudogenitalium, Corynebacterium urealyticum, Enterobacter cloacae complex, Enterococcus raffinosus, Epstein-Barr virus (EBV), Facklamia hominis, Finegoldia magna (Peptostreptococcus magnus), Human adenovirus B, Human papillomavirus type 51 (HPV 51; High-risk), Human papillomavirus type 55/44 (HPV 55/44; Low-risk), Human papillomavirus type 56 (HPV 56; High-risk), Human papillomavirus type 68 (HPV 68; High-risk), JC polyomavirus, Klebsiella aerogenes (Enterobacter aerogenes), Klebsiella quasipneumoniae, Klebsiella variicola, Mobiluncus curtisii, Oligella urethralis, Peptostreptococcus anaerobius, Porphyromonas asaccharolytica, Prevotella timonensis, Propionimicrobium lymphophilum, Providencia stuartii, Rothia kristinae (Kocuria kristinae), Salmonella enterica, Serratia marcescens, Staphylococcus haemolyticus, Staphylococcus hominis, Staphylococcus simulans, Staphylococcus warneri, Streptococcus anginosus, Streptococcus constellatus, Streptococcus intermedius, Trichomonas vaginalis, Ureaplasma parvum
Unique to Culture	16	Enterococcus faecalis, Acinetobacter baumannii, Enterobacter aerogenes, Enterobacter cloacae, Enterobacter cloacae, Enterococcus faecalis, *E. coli, E. coli*, Klebsiella pneumoniae, Klebsiella oxytoca, Klebsiella oxytoca, Lactobacillus species, Proteus mirabilis, Staphylococcus aureus
Unique to PCR	14	Actinotignum schaalii, Candida albicans, Candida glabrata, Candida parapsilosis, Citrobacter freundii/braakii/koseri, *E. coli* (E. coli), Enterobacter cloaca, Enterobacter cloacae, Enterococcus faecium, Klebsiella aerogenes, Klebsiella pneumoniae, Prevotella bivia, Streptococcus agalactiae
Overlap: All Three Methods	3	*Enterococcus faecalis, E. coli, Proteus mirabilis*
Overlap: mNGS + PCR	8	Bacteroides fragilis, Enterococcus faecium, Klebsiella oxytoca, Klebsiella pneumoniae, Morganella morganii, Pseudomonas aeruginosa, Staphylococcus aureus, Streptococcus agalactiae
Overlap: mNGS + Culture	1	*Staphylococcus epidermidis*
Overlap: Culture + PCR	4	Citrobacter freundii, Enterococcus faecalis, Enterococcus faecium, *E. coli*

While precision metagenomics demonstrated superior sensitivity and the ability to detect a broader range of organisms compared to traditional methods, some limitations were observed. precision metagenomics occasionally struggled to differentiate between closely related species, such as *Prevotella timonensis* and *P. bivia*. Additionally, the implementation of precision metagenomics in clinical settings may be hindered by its higher cost and the need for specialized bioinformatic expertise to interpret the results. Further studies are recommended to optimize this approach and address these limitations.

## Ethics Statement

Ethical review and approval were not required for the study on human participants in accordance with the local legislation and institutional requirements. The patients/participants provided their written informed consent to participate in this study. This research aligns with the guidelines for exemption from human subjects’ research oversight as outlined by regulatory bodies, including the U.S. Department of Health and Human Services (45 CFR 46).

## CRediT Author Statement

**Rob E. Carpenter:** Conceptualization, Writing-reviewing and editing; **Sadia Almas:** Study design and execution; **Vaibhav K. Tamrakar:** Data curation and SRA submission; **Rahul Sharma:** Conceptualization.

## Data Availability

NCBIPRJNA986135 (Original data).

## Appendix Supplementary materials

Image, application 1

## Footnotes

1 The Explify® bioinformatics analysis was limited to qualitative detection because of the comparative methods (culture and qualitative PCR) used in the study. The Explify® platform is capable of reporting absolute abundance (organism/ml) of organisms in clinical specimens derived from the RPKM value of a known quantity of spiked T7 Phase. Quantitative analysis was beyond this study. See https://www.illumina.com/products/by-type/informatics-products/basespace-sequence-hub/apps/explify-upip-data-analysis.html
